# Lack of Additive Effects of Resveratrol and Energy Restriction in the Treatment of Hepatic Steatosis in Rats

**DOI:** 10.3390/nu9070737

**Published:** 2017-07-11

**Authors:** Iñaki Milton-Laskibar, Leixuri Aguirre, Alfredo Fernández-Quintela, Anabela P. Rolo, João Soeiro Teodoro, Carlos M. Palmeira, María P. Portillo

**Affiliations:** 1 Nutrition and Obesity Group, Department of Nutrition and Food Science, University of the Basque Country (UPV/EHU) and Lucio Lascaray Research Institute, Facultad de Farmacia, Vitoria 01006, Spain; inaki.milton@ehu.eus (I.M.-L.); leixuri.aguirre@ehu.eus (L.A.); alfredo.fernandez@ehu.eus (A.F.-Q.); 2 CIBERobn Physiopathology of Obesity and Nutrition, Institute of Health Carlos III, Vitoria 01006, Spain; 3 Department of Life Sciences and Center for Neurosciences and Cell Biology, University of Coimbra, Coimbra 3004-517, Portugal; anpiro@ci.uc.pt (A.P.R.); jteodoro@ci.uc.pt (J.S.T.); palmeira@ci.uc.pt (C.M.P.)

**Keywords:** resveratrol, energy restriction, liver steatosis, rat

## Abstract

The aims of the present study were to analyze the effect of resveratrol on liver steatosis in obese rats, to compare the effects induced by resveratrol and energy restriction and to research potential additive effects. Rats were initially fed a high-fat high-sucrose diet for six weeks and then allocated in four experimental groups fed a standard diet: a control group, a resveratrol-treated group, an energy restricted group and a group submitted to energy restriction and treated with resveratrol. We measured liver triacylglycerols, transaminases, FAS, MTP, CPT1a, CS, COX, SDH and ATP synthase activities, FATP2/FATP5, DGAT2, PPARα, SIRT1, UCP2 protein expressions, ACC and AMPK phosphorylation and PGC1α deacetylation. Resveratrol reduced triacylglycerols compared with the controls, although this reduction was lower than that induced by energy restriction. The mechanisms of action were different. Both decreased protein expression of fatty acid transporters, thus suggesting reduced fatty acid uptake from blood stream and liver triacylglycerol delivery, but only energy restriction reduced the assembly. These results show that resveratrol is useful for liver steatosis treatment within a balanced diet, although its effectiveness is lower than that of energy restriction. However, resveratrol is unable to increase the reduction in triacylglycerol content induced by energy restriction.

## 1. Introduction

Excessive fat accumulation in the liver is known as simple hepatic steatosis, which is the most benign form of non-alcoholic fatty liver disease (NAFLD). It is a major cause of chronic liver disease in western societies, and this burden is expected to grow with the increasing incidence of obesity and metabolic syndrome, which are both closely associated with it [1,2]. Energy restriction is a commonly used method for fatty liver treatment [3,4]. In fact, this method has been proved to induce a decrease in intrahepatic fat content in overweight and obese subjects [5,6].

A great deal of attention has been paid by the scientific community in recent years to bioactive molecules present in foods and plants, such as phenolic compounds, which could represent new complementary tools for liver steatosis management. One of the most widely studied molecules is resveratrol (*trans*-3,5,4′-trihydroxystilbene), a phytoalexin occurring naturally in grapes, berries and peanuts [7,8]. Numerous studies have been carried out using resveratrol and different models of liver steatosis in mice and rats [9,10]. The vast majority of these studies have demonstrated that resveratrol is able to prevent liver triacylglycerol accumulation induced by overfeeding conditions. With regard to human beings, its positive effects on liver steatosis have been observed in studies carried out by its administration at doses in the range of 150–500 mg/day for 4–12 weeks [9,11,12,13]. Nevertheless, it is important to point out that other authors have not observed this beneficial effect [14].

Furthermore, it has been proposed that resveratrol may mimic energy restriction in rodent models [15,16,17,18]. Thus, this compound could bring about the benefits of energy restriction without an actual reduction in calorie intake.

Taking all of the information above into account, the aims of the present study were (a) to analyze the effect of resveratrol on liver steatosis previously induced by a high-fat high-sucrose diet in obese rats; (b) to compare the effects induced by resveratrol and energy restriction and (c) to research potential additive effects between resveratrol and energy restriction. Our initial hypothesis is that resveratrol can show a delipidating effect in the liver similar to that induced by a mild energy restriction, and that the combination of both strategies can increase treatment effectiveness.

## 2. Material and Methods

### 2.1. Animals, Diets and Experimental Design

The experiment was conducted with forty five 6-week-old male Wistar rats from Harlan Ibérica (Barcelona, Spain) and performed in accordance with the institution guide for the care and use of laboratory animals (M20_2016_039).

The rats were individually housed in polycarbonate metabolic cages (Tecniplast Gazzada, Buguggiate, Italy) and placed in an air-conditioned room (22 ± 2 °C) with a 12-h light-dark cycle. After a 6-day adaptation period, all rats were fed a high-fat high-sucrose (HFHS) diet (OpenSource Diets, Lynge, Denmark; Ref. D12451), for six weeks. This diet provided 45% of the energy as fat, 20% as protein and 35% as carbohydrates (4.7 kcal/g diet). After this period, nine rats (HFHS group) were sacrificed to check whether liver steatosis was induced by comparing their liver lipid content with that of a matched group of rats fed a standard diet for six weeks (normal rats; N group). The remaining animals fed the high-fat high-sucrose diet for six weeks were randomly divided into four experimental groups (*n* = 9): the control group (C), the resveratrol group treated with resveratrol (RSV), the restricted group submitted to a moderate energy restriction (R), and the group both treated with resveratrol as well as submitted to energy restriction (RR). In all cases, the diet was a semi-purified standard diet (OpenSource Diets, Lynge, Denmark; D10012G), and the additional treatment period was six weeks. This semi-purified standard diet provided 16% of the energy as fat, 20% as protein and 64% as carbohydrates (3.9 kcal/g diet). Rats from C and RSV groups had free access to food, and rats from R and RR groups were subjected to a 15% energy restriction. This percentage, that was selected according to previous studies from our laboratory, is below the percentage commonly used in energy restricted diets in humans. The diet amount provided to the rats on the restricted groups was calculated based on the spontaneous food intake in C group. In the RSV and RR groups, resveratrol was added to the diet as previously reported [8] to ensure a dose of 30 mg/kg body weight/day.

At the end of the total experimental period (12 weeks), rats from the four experimental groups were sacrificed after 8–12 h of fasting, under anesthesia (chloral hydrate), by cardiac exsanguination. Livers were dissected, weighed and immediately frozen in liquid nitrogen. Serum was obtained from blood samples after centrifugation (1000× *g* for 10 min, at 4 °C). All samples were stored at −80 °C until analysis.

### 2.2. Liver Triacylglycerol Content and Serum Transaminases

Total liver lipids were extracted according to the method described by Folch et al. [19]. The lipid extract was dissolved in isopropanol, and the triacylglycerol content was measured using a commercial kit (Spinreact, Barcelona, Spain). Commercial kits were also used for the analysis of serum transaminases aspartate aminotransferase (AST) and alanine aminotransferase (ALT) (Spinreact, Barcelona, Spain).

### 2.3. Enzyme Activities

The activity of the lipogenic enzyme fatty acid synthase (FAS) was measured by spectrophotometry, as previously described [20]. Briefly, liver samples (0.5 g) were homogenized in 5 mL of buffer (150 mM KCl, 1 mM MgCl_2_, 10 mM *N*-acetyl cysteine and 0.5 mM dithiothreitol) and centrifugated to 100,000× *g* for 40 min at 4 °C. The supernatant fraction was used for FAS activity determination, as the rate of malonyl CoA dependent NADH oxidation [21]. Results were expressed as nanomoles of reduced nicotinamide adenine dinucleotide phosphate (NADPH) consumed per minute per milligram of protein.

In order to assess the assembly and secretion of very low density lipoproteins by the liver, microsomal triglyceride transfer protein (MTP) activity was determined fluorimetrically by using a commercial kit (Sigma-Aldrich, St. Louis, MI, USA). MTP activity was expressed as percentage of transference.

As far as oxidative enzymes are concerned, carnitine palmitoyltransferase-1a (CPT-1a) activity was measured spectrophotometrically in the mitochondrial fraction as previously described [22]. The activity was expressed as nanomoles of coenzyme A formed per minute per milligram of protein. Citrate synthase (CS) activity was assessed spectrophotometrically following the Srere method [23], by measuring the appearance of free CoA. Briefly, frozen liver samples were homogenized in 25 vol (wt/vol) of 0.1 M Tris-HCl buffer (pH 8.0). Homogenates were incubated for 2 min at 30 °C with 0.1 M Tris-HCl buffer containing 0.1 mM DTNB, 0.25 Triton *X*-100, 0.5 mM oxalacetate and 0.3 mM acetyl CoA, and readings were taken at 412 nm. Then, the homogenates were re-incubated for 5 min and readings were taken at the same wavelength. CS activity was expressed as CoA nanomoles formed per minute, per milligram of protein. The protein content of the samples was determined by the [24], using bovine serum albumine as standard.

For succinate dehydrogenase (SDH), cytochrome *c* oxidase (COX) and mitochondrial ATP synthase activity determinations, liver samples were powdered with liquid nitrogen, using a mortar and a pestle, and homogenized with homogenization buffer (250 mM sucrose, 10 mM HEPES (pH 7.4), 0.5 mM EGTA and 0.1% fat-free bovine serum albumin) using a Ystral D-79282 homogenizer (Ystral, Ballrechten-Dottingen, Germany). The protein content of the samples was determined using the Biuret method [25], and calibrated with bovine serum albumin. SDH activity was determined polarographically as previously described [26]. Briefly, liver homogenates (2 mg of protein) were suspended under constant magnetic stirring at 25 °C, in 1.4 mL of standard respiratory medium (130 mM sucrose, 50 mM KCl, 5 mM MgCl_2_, 5 mM KH_2_PO_4_, 50 µM EDTA and 5 mM HEPES (pH 7.4) supplemented with 5 mM succinate, 2 μM rotenone, 0.1 μg Antimycin A, 1 mM KCN and 0.3 mg Triton *X*-100. The reaction was initiated by the addition of 1 mM phenazine methosulfate (PMS). In the case of COX, the activity was also measured polarographically, as previously described [27]. The reaction was carried out at 25 °C in 1.4 mL of standard respiratory medium, supplemented with 2 µM rotenone, 10 µM oxidized Cytochrome *c* and 0.3 mg Triton *X*-100. After the addition of 2 mg of liver homogenate protein, the reaction was initiated by adding 5 mM ascorbate plus 0.25 mM tetra methylphenylene-diamine (TMPD). Finally, the activity of ATP Synthase was determined spectrophotometrically at a wavelength of 660 nm, in association with ATP hydrolysis as previously mentioned [28]. Briefly, 2 mg of liver homogenate protein were incubated with 2 mL of reaction medium (125 mM sucrose, 65 mM KCl, 2.5 mM MgCl_2_ and 0.5 mM HEPES, pH 7.4) at 37 °C. The reaction was initiated by adding 2 mM Mg^2+^-ATP in the presence or absence of oligomicyn (1 µg/mg protein), and stopped after 3 min by adding 1 mL of 40% trichloroacetic acid. The samples were then centrifugated for 5 min at 3000 rpm, and 1 mL of the supernatant was mixed with 2 mL of H_2_O and 2 mL of ammonium molybdate. The ATP synthase activity was calculated as the difference in total absorbance and absorbance in the presence of oligomycin.

### 2.4. Western Blot

For Acetyl CoA carboxylase (ACC), AMP activated protein kinase (AMPK α), sirtuin 1 (SIRT1), fatty acid transport protein 2 (FATP2), uncoupling protein 2 (UCP2), diacylglycerol acyltransferase 2 (DGAT2), fatty acid transport protein 5 (FATP5) and β-actin protein quantification, liver samples of 100 mg were homogenated in 1000 μL of cellular PBS (pH 7.4), containing protease inhibitors (100 mM phenylmethylsulfonyl fluoride and 100 mM iodoacetamide). Homogenates were centrifuged at 800× *g* for 10 min at 4 °C. Protein concentration in homogenates was measured by the Bradford method [24] using bovine serum albumin as standard. In the case of peroxisome proliferator-activator receptor alpha (PPARα), and peroxisome proliferator-activated receptor gamma coactivator 1-alpha (PGC1α), nuclear protein extraction was carried out with 100 mg of liver tissue, as previously described [29].

Immunoblot analyses were performed using 60 μg of protein from total or nuclear liver extracts separated by electrophoresis in 7.5% or 10% SDS-polyacrylamide gels and transferred to PVDF membranes. The membranes were then blocked with 5% casein PBS-Tween buffer for 2 h at room temperature. Subsequently, they were blotted with the appropriate antibodies overnight at 4 °C. Protein levels were detected via specific antibodies for ACC (1:1000), AMPK α (1:1000) (Cell Signaling Technology, Danvers, MA, USA), SIRT1 (1:1000), FATP2 (1:1000), UCP2 (1:500), DGAT2 (1:500) (Santa Cruz Biotech, Dallas, TX, USA) FATP5 (1:500), (LifeSpan BioScience, Seattle, WA, USA), PGC1α (1:1000), PPARα (1:500), (Abcam, Cambridge, UK) and β-actin (1:5000) (Sigma, St. Louis, MO, USA). Afterward, polyclonal anti-mouse for β-actin, anti-rabbit for ACC, AMPK, SIRT1, DGAT2, FATP5, PGC1α and PPARα, and anti-goat for FATP2 and UCP2 (1:5000) were incubated for 2 h at room temperature, and ACC, AMPK, SIRT1, FATP2, UCP2, DGAT2, PPARα, FATP5, PGC1α and β-actin were measured. After antibody stripping, the membranes were blocked, and then incubated with phosphorylated ACC (serine 79, 1:1000), phosphorylated AMPK (threonine 172, 1:500) and acetylated lysine (1:1000) (Cell Signaling Technology, Danvers, MA, USA) antibodies. The bound antibodies were visualized by an ECL system (Thermo Fisher Scientific Inc., Rockford, IL, USA) and quantified by a ChemiDoc MP Imaging System (Bio-Rad, Hercules, CA, USA). The measurements were normalized by β-actin in total protein extractions and in the case of the nuclear extraction, equal loading of proteins was confirmed by staining the membranes with Comassie Blue.

### 2.5. Statistical Analysis

Results are presented as mean ± SEM. Statistical analysis was performed using SPSS 24.0 (SPSS, Chicago, IL, USA). All the parameters are normally-distributed according to the Shapiro-Wilks test. Data were analyzed by one-way ANOVA followed by Newman-Keuls post-hoc test. Significance was assessed at the *p* < 0.05 level.

## 3. Results

### 3.1. Body Weight Gain, Liver Weight, Liver Triacylglycerol Amounts and Serum Transaminases

As explained in the Results section, after six weeks of high-fat high-sucrose feeding, rats (HFHS group) showed significantly increased amounts of triacylglycerols in their livers than rats fed a standard diet for six weeks (N group) (53.6 ± 1.9 mg/g tissue vs. 32.6 ± 4.1 mg/g tissue; *p* < 0.050), indicating that liver steatosis was induced. These results were paralleled by the induction of insulin resistance, as observed in a previous study from our laboratory carried out in this cohort of rats [30].

Body weight gain was similar in C and RSV groups and lower in both restricted groups when compared with the C group (*p* < 0.0003 in R group and *p* < 0.0001 in RR group), with no difference between them. In spite of this difference between restricted and non restricted groups, no differences were observed in liver weight among the four experimental groups (Table 1).

Lower values of triacylglycerol content were found in the three treated groups in comparison with the C group (*p* < 0.03 in RSV group, *p* < 0.0002 in R group and *p* < 0.0004 in RR group). In the case of the groups submitted to a mild energy restriction (R and RR), lower values were found compared with the RSV group (*p* < 0.003 in R group and *p* < 0.005 in RR group), with no differences between them (Table 1).

As far as serum parameters are concerned, triacylglycerols were not modified in resveratrol-treated rats when compared with control animals. By contrast, restricted rats (R and RR groups) showed significantly lower values without differences between them. No changes in serum transaminase concentrations were observed among experimental groups (Table 1).

### 3.2. Enzyme Activities

**Figure 1:**
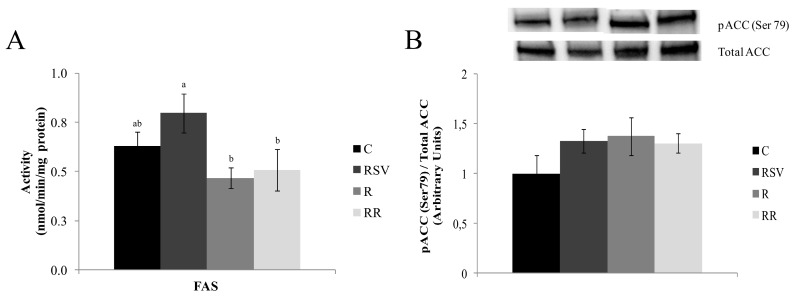
FAS activity (**A**) and phosphorylated ACC (serine 79)/Total ACC ratio (**B**) in liver from rats fed an obesogenic diet for six weeks, and then fed a standard diet (C), or a standard diet supplemented with resveratrol (RSV), or submitted to energy restriction and fed a standard diet (R) or submitted to energy restriction and fed a standard diet supplemented with resveratrol (RR) (*n* = 9/group) for additional six weeks. Values are mean ± SEM. Differences among groups were determined by using one-way ANOVA followed by Newman Keuls post-hoc test. Values not sharing a common letter are significantly different (*p* < 0.05). FAS: fatty acid synthase, ACC: acetyl CoA carboxylase.

**Figure 2:**
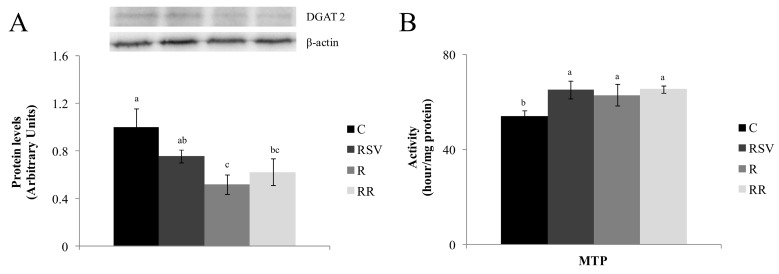
DGAT2 (**A**) protein expression and MTP (**B**) activity in liver from rats fed an obesogenic diet for six weeks, and then fed a standard diet (C), or a standard diet supplemented with resveratrol (RSV), or submitted to energy restriction and fed a standard diet (R) or submitted to energy restriction and fed a standard diet supplemented with resveratrol (RR) (*n* = 9/group) for additional six weeks. Values are mean ± SEM. Differences among groups were determined by using one-way ANOVA followed by Newman Keuls post-hoc test. Values not sharing a common letter are significantly different (*p* < 0.05). DGAT2: diacylglycerol acyltransferase 2, MTP: microsomal triglyceride transfer protein.

No differences in FAS activity were found between the control and each treated group (Figure 1A). On the other hand, MTP activity was greater in the three treated groups when compared with the C group (*p* < 0.016 in RSV group, *p* < 0.05 in R group and *p* < 0.0016 in RR group), without significant differences among the three (Figure 2B).

**Figure 3:**
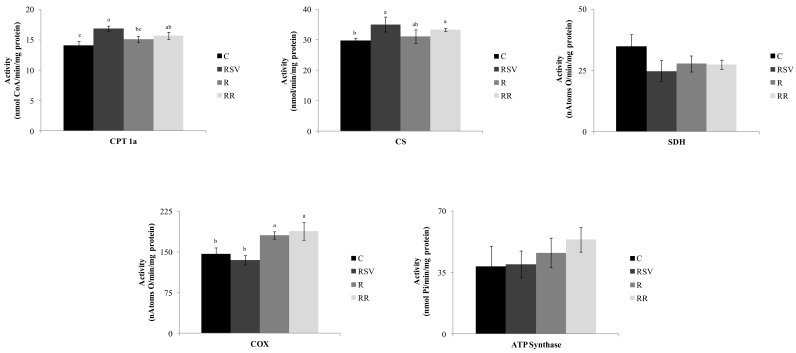
CPT1 and CS, SDH, COX and ATP Synthase activities in liver from rats fed an obesogenic diet for six weeks, and then fed a standard diet (C), or a standard diet supplemented with resveratrol (RSV), or submitted to energy restriction and fed a standard diet (R) or submitted to energy restriction and fed a standard diet supplemented with resveratrol (RR) (*n* = 9/group) for additional six weeks. Values are mean ± SEM. Differences among groups were determined by using one-way ANOVA followed by Newman Keuls post-hoc test. Values not sharing a common letter are significantly different (*p* < 0.05). CPT1a: carnitine palmitoyltransferase-1a, CS: citrate synthase, SDH: succinate dehydrogenase, COX: cytochrome *c* oxidase.

With regard to oxidative enzymes, the activity of CPT1a was increased in the groups supplemented with resveratrol when compared with the C group (*p* < 0.002 in RSV group and *p* < 0.05 in RR group), with no difference between them. A significantly higher enzyme activity was also observed in the RSV (*p* < 0.01) group when compared with the R group (Figure 3). In the case of the CS activity, the RSV and RR groups showed greater activity when compared with the C group (*p* < 0.03 and *p* < 0.003 respectively), with no differences between them (Figure 3). Moreover, no differences were observed in SDH, (also known as respiratory Complex II) or ATP synthase among experimental groups (Figure 3). Finally, the activity of mitochondrial Complex IV (COX) was significantly increased in both restricted groups (*p* < 0.01 and *p* < 0.01 in R and RR groups respectively), with no differences between them. Its activity in resveratrol-treated rats remained unchanged when compared with the control group (Figure 3).

### 3.3. Western Blot Analysis

**Figure 4:**
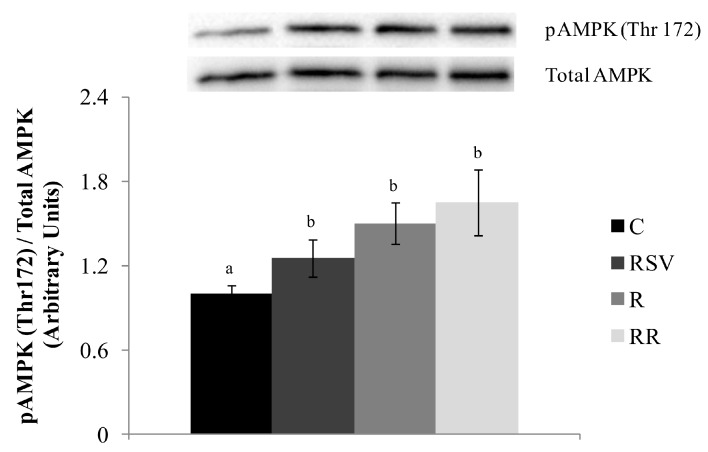
Phosphorylated AMPK (threonine 172)/Total AMPK ratio in liver from rats fed an obesogenic diet for six weeks, and then fed a standard diet (C), or a standard diet supplemented with resveratrol (RSV), or submitted to energy restriction and fed a standard diet (R) or submitted to energy restriction and fed a standard diet supplemented with resveratrol (RR) (*n* = 9/group) for additional six weeks. Values are mean ± SEM. Differences among groups were determined by using one-way ANOVA followed by Newman Keuls post-hoc test. Values not sharing a common letter are significantly different (*p* < 0.05). AMPK: AMP activated protein kinase.

The ratio pACC (Ser 79)/Total ACC was used as an index of ACC activity. High values of this ratio were found in treated groups when compared with the controls (+33% in RSV group, +37% in R group and +30% in RR group). These differences showed a statistical trend (*p* = 0.08) (Figure 1B). In the case of pAMPKα (Thr 172)/Total AMPKα ratio, which shows the activation of this enzyme, the three treated groups showed greater phosphorylation (*p* < 0.05 in RSV group, *p* < 0.005 in R group and *p* < 0.01 in RR group), which is to say activation, when compared with C group, with no differences among the three (Figure 4). 

**Figure 5:**
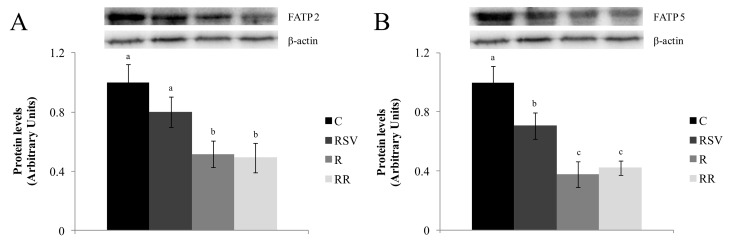
FATP2 (**A**) and FATP5 (**B**) protein expression in liver from rats fed an obesogenic diet for six weeks, and then fed a standard diet (C), or a standard diet supplemented with resveratrol (RSV), or submitted to energy restriction and fed a standard diet (R) or submitted to energy restriction and fed a standard diet supplemented with resveratrol (RR) (*n* = 9/group) for additional six weeks. Values are mean ± SEM. Differences among groups were determined by using one-way ANOVA followed by Newman Keuls post-hoc test. Values not sharing a common letter are significantly different (*p* < 0.05). FATP2: fatty acid transport protein 2, FATP5: fatty acid transport protein 5.

DGAT2 was also measured and lower protein expression was showed by rats from the restricted groups when compared with the C group (*p <* 0.01 in R group and *p <* 0.04 in RR group), with no differences between them (Figure 2B). As far as FATP2 protein is concerned, the groups submitted to a mild energy restriction showed the lowest values, in comparison with the C group (*p* < 0.003 in R group and *p* < 0.003 in RR group), with no difference between them (Figure 5A). On the other hand, in all the treated groups FATP5 protein expression was lower than that in the C group (*p* < 0.03 in RSV group, *p* < 0.0003 in R group and *p* < 0.0004 in RR group) (Figure 5B).

**Figure 6:**
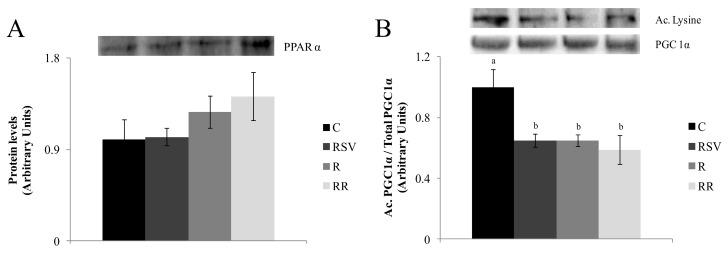
PPARα protein expression (**A**) and Acetylated PGC1α/Total PGC1α (**B**) in liver from rats fed an obesogenic diet for six weeks, and then fed a standard diet (C), or a standard diet supplemented with resveratrol (RSV), or submitted to energy restriction and fed a standard diet (R) or submitted to energy restriction and fed a standard diet supplemented with resveratrol (RR) (*n* = 9/group) for additional six weeks. Values are mean ± SEM. Differences among groups were determined by using one-way ANOVA followed by Newman Keuls post-hoc test. Values not sharing a common letter are significantly different (*p* < 0.05). PPARα: peroxisome proliferator-activator receptor alpha, PGC1α: peroxisome proliferator-activated receptor gamma coactivator 1-alpha.

**Figure 7:**
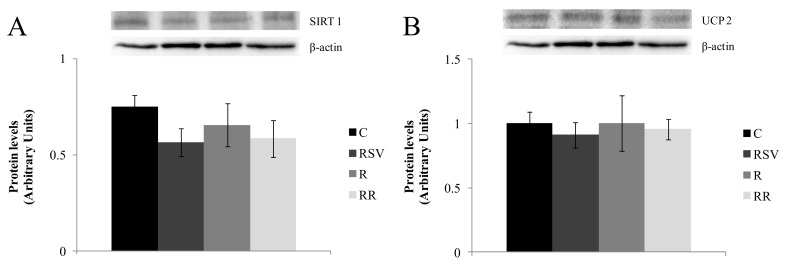
SIRT1 (**A**) and UCP2 (**B**) protein expression in liver from rats fed an obesogenic diet for six weeks and then fed a standard diet (C), or a standard diet supplemented with resveratrol (RSV), or submitted to energy restriction and fed a standard diet (R) or submitted to energy restriction and fed a standard diet supplemented with resveratrol (RR) (*n* = 9/group) for additional six weeks. Values are mean ± SEM. Differences among groups were determined by using one-way ANOVA followed by Newman Keuls post-hoc test. Values not sharing a common letter are significantly different (*p* < 0.05). SIRT1: sirtuin 1, UCP2: uncoupling protein 2.

Regarding proteins related to fatty acid oxidation, no significant changes were induced by experimental treatments in the expression of PPARα (Figure 6A). In the case of PGC-1α acetylation, reduced levels were observed in all treated groups (*p* < 0.01 in RSV group, *p* < 0.01 in R group and *p* < 0.008 in RR group), with no differences among them (Figure 6B). Finally, when the protein expression of SIRT1 and UCP2 were studied, no changes were observed among the different groups (Figure 7).

## 4. Discussion

The effectiveness of resveratrol in the reduction of hepatic lipid accumulation, when administered under overfeeding conditions and concurrent with an obesogenic diet, has been largely reported in rodents in the prevention of steatosis [31,32,33,34,35,36,37,38,39,40]. Indeed, resveratrol is able to partially prevent liver steatosis associated with overfeeding. However, much less abundant information is available concerning its effects on previously developed liver steatosis reduction [41]. Bearing this in mind, and taking into account that it has been proposed that resveratrol mimics energy restriction [11,16,17,42], which is a common dietary strategy for steatosis treatment, the first aim of the present study was to analyze the effect of this compound on liver steatosis. This had been previously induced by an obesogenic diet when it was added to a standard diet. In the present study, a dose of 30 mg resveratrol/kg body weight/day was used because in a previous study we observed that it was an effective dose in reducing liver triacylglycerol amount in an overfeeding model [35].

For this purpose, the C and RSV groups were compared. The lower hepatic triacylglycerol content observed in the rats from the RSV group (−23.4%) showed that resveratrol was indeed effective, not only in preventing steatosis, as widely described in literature, but also in reducing fat accumulation previously induced by a high-fat high-sucrose feeding. When we compare the percentage of triacylglycerol reduction obtained in this study (−23.4%) with that found in a previous study from our group that was devoted to analyzing the preventive effect of resveratrol on liver steatosis and carried out with the same dose of resveratrol and the same experimental period length (−23.0%) [35], it can be observed that the effectiveness of resveratrol as a preventative molecule was only slightly higher than it was as a therapeutic one. This conclusion is not in good accordance with that obtained by Heebøll et al., who found that the preventive effect of resveratrol was superior to its therapeutic effect. This discrepancy may be due to differences in the experimental design (mainly animal species and resveratrol dose). Surprisingly, serum transaminases were not reduced. This lack of effect may have been due to their being in the range of physiological values [43] after six weeks of obesogenic feeding, as a consequence of the development of a mild degree of steatosis.

Insulin resistance is closely related to liver steatosis. This alteration in glucose homeostasis was studied in this cohort of rats in a previous paper [30], by measuring serum insulin and glucose, HOMA-IR and by carrying out a glucose tolerance test. We observed that resveratrol induced a mild improvement in glycemic control, which fits well with the reduction observed in liver steatosis in these rats.

The amount of triacylglycerols accumulated in hepatocytes is regulated by various metabolic processes: fatty acid uptake, fatty acid synthesis and triacylglycerol esterification on the one hand (“input”), and fatty acid oxidation and triacylglycerol export on the other hand (“output”). Steatosis occurs when “input” exceeds “output” [44,45]. In order to analyze the mechanisms underlying the delipidating action of resveratrol, we assessed its effects on several parameters related to the previously mentioned processes.

As far as de novo lipogenesis in concerned, although FAS activity remained unchanged, a sharp increase in the activity of ACC, the limiting enzyme of this process, was observed in resveratrol-treated rats. Consequently, it can be proposed that this metabolic pathway was likely somehow inhibited by this polyphenol, and thus this could contribute to the reduction in triacylglycerol content. Moreover, FATP5 protein expression was reduced in the RSV group, suggesting a decrease in fatty acid uptake, which could also contribute to the reduction in triacylglycerol content. In fact, the relationship between FATP5 and NAFLD development has been studied in rodents [46] and in humans [47]. As far as fatty acid oxidation is concerned, its involvement in liver delipidation is not clear. Thus, the activities of CPT1a, the enzyme that allows long chain fatty acids to enter into mitochondria, and CS, a marker of mitochondria density, were significantly increased due to resveratrol treatment; this was also the case for the deacetylation level of PGC-1α, the transcription factor co-activator that regulates mitochondria number and function [48,49]. By contrast, the activities of enzymes participating in the respiratory electron transport chain, SDH, COX, and ATP synthase remained unchanged.

DGAT2, the enzyme that catalyzes the binding between diacylglycerol and a long chain fatty acyl-CoA, was not modified by resveratrol treatment. This suggests that the synthesis of triacylglycerols could be reduced by a decrease in fatty acid availability, but not by the inhibition of the assembly process. Moreover, increased MTP activity suggests enhanced delivery of triacylglycerols from liver to plasma. In spite of this effect, serum triacylglycerol concentration was not increased. In order to explain this fact, it is important to remember that this parameter depends not only on triacylglycerol delivery to blood, but also on triacylglycerol clearance from tissues. Thus, increased triacylglycerol clearance in skeletal muscle via lipoprotein lipase cannot be discarded. Although there are no reports in the literature showing the effect of resveratrol on skeletal muscle LPL, our hypothesis stems from the fact that Timmers et al., [11] proposed that resveratrol mimics the effects of training in skeletal muscle, and by the reported increase in LPL expression induced by training in skeletal muscle [50,51].

Taken together, these results suggest that the reduction in hepatic triacylglycerols induced by resveratrol is mainly justified by decreased fatty acid availability for triacylglycerol synthesis, due to reduced de novo synthesis and uptake and increased oxidation, and to the increase in triacylglycerol delivery to blood. 

The role of UCP2 in NAFLD development has been intensively studied, but reported studies are controversial [52]. Some studies have shown that hepatocellular UCP2 expression is increased in NAFLD, indicating its potential role in disease development [53,54,55,56]. However, other studies have demonstrated that UCP2 deficiency caused diminished hepatic utilization and fatty acid clearance and thus may lead to liver steatosis [57]. Moreover, it has been reported that obesity-related fatty liver is unchanged in UCP2 mitochondrial-deficient mice [55]. Thus, in the present study we analyzed UCP2 protein expression in order to gain more insight concerning this issue. Unfortunately, no changes were observed after resveratrol treatment, meaning that irrespective of the positive or negative effect of UCP2 on steatosis, the delipidating effect of this phenolic compound was not mediated by this uncoupling protein. This result agrees with that reported by Heebøll et al. in mice [41].

Resveratrol has been identified as a potent activator for both SIRT1 and AMPK, two critical signalling molecules regulating the pathways of hepatic lipid metabolism [58]. In the present study, AMPK phosphorylation was increased in the RSV group, meaning that this enzyme was activated by the polyphenol treatment. As far as SIRT1 is concerned, although its protein expression was not modified, the increased deacetylation level of PGC-1α, one of its main targets, suggests that this deacetylase was activated by resveratrol. Consequently, it can be stated that, under our experimental conditions, the activation of the axis SIRT1/AMPK was also involved in resveratrol-induced effects.

Although, as stated in this paper’s discussion section, resveratrol is considered an energy restriction mimetic, several authors who have analyzed actions of this polyphenol other than on fatty liver have proposed that the mechanisms underlying the effects of resveratrol and energy restriction are not always the same [17,30,59,60]. In this context, a second aim of the present study was to compare the effects of a mild energy restriction and resveratrol on liver steatosis. Rats from the R group showed a significant reduction in hepatic triacylglycerol when compared with the control group (−56.3%). De novo lipogenesis seems to be reduced in the restricted group because the activity of ACC was decreased by 37%. Furthermore, fatty acid uptake was reduced, as shown by the decrease in FATP2 and FATP5. With regard to the potential contribution of fatty acid oxidation pathway, the results show that energy restriction increased activation of PGC-1α and the activity of COX, with no changes in the rest of oxidative parameters. These results are not surprising because Nisoli et al. [61] reported that a 30% calorie restriction on mice for three months resulted in greatly increased liver mitochondria, evidenced by increases in the proteins cytochrome *c* and cytochrome oxidase subunit IV, and the mRNA levels of PGC-1α, among others. These findings have led to the general acceptance, and have led to incorporation of the concept that energy restriction induces mitochondrial biogenesis. However, Hancock et al. [62] did not find any change in mitochondrial markers in the liver after 14 weeks of 30% energy restriction.

Moreover, the reduced amount of DGAT2 in the R group suggests a decrease in triacylglycerol assembly. These results show that a decrease in triacylglycerol synthesis, due to reduced availability of one of the substrates (fatty acids) and the inhibition of the assembly process, contributed to the reduction in hepatic triacylglycerol content induced by energy restriction. Finally, increased MTP activity indicates enhanced triacylglycerol delivery from liver to plasma. In spite of this effect, serum concentration of triacylglycerols was lower in the R and C group. As in the case of resveratrol treated-rats, it can be argued that due to energy restriction, other tissues can increase the uptake of this lipid species via lipoprotein lipase [63].

As expected, AMPK was phosphorylated and thus, activated. On the other hand, protein expression of SIRT1 was not modified. However, the deacetylation status of PGC-1α suggests its activation. Consequently, it can be stated that under the activation of the axis SIRT1/AMPK was involved in the delipidating effect induced by a mild energy restriction effects.

By comparing the RSV and R groups it can be observed that hepatic fat reduction induced by energy restriction was greater than that induced by resveratrol treatment, meaning that a mild energy restriction (−15%) was more efficient than resveratrol administration. Similarly, the improvement in glycemic control observed in this cohort of rats in our previous paper mentioned before in this paper’s discussion section, was greater than that observed in rats treated with resveratrol [30]. In addition, the mechanisms of action of resveratrol and energy restriction were not exactly the same. Both treatment strategies decreased de novo lipogenesis, fatty acid uptake from blood stream and increased fatty acid oxidation and liver triacylglycerol delivery, but only energy restriction reduced triacylglycerol assembly. These results are in good accordance with those reported by Tauriainen et al. [33] when they analyzed the preventive effects of resveratrol and energy restriction on liver steatosis under overfeeding conditions. These authors observed that whereas energy restriction (−30%) totally prevented liver steatosis associated to obesogenic feeding, resveratrol only prevented it partially.

Finally, a third aim of the present study was to seek the effects of resveratrol under energy restriction conditions, and to search for potential additive effects between both treatments. This being the case, the administration or resveratrol together with a restricted diet would increase the effectiveness of this dietary treatment. At this point, it is important to emphasize that although in the vast majority of the reported studies the energy restriction ranges from 20% to 40%, in this case, a lower degree of restriction was chosen (15%) was chosen in the present study. The reason for this was based on a previous study from our group [64]. In that study, we also looked for additive anti-obesity and anti-diabetic effects between resveratrol, at a dose of 30 mg/kg of body weight/day, and 25% energy restriction. We observed that the addition of resveratrol to the restricted diet did not lead to additional reductions in fat mass or in serum insulin concentrations with regard to those produced by energy restriction alone. We believed that one of the reasons that could explain this situation was that the effects caused by energy restriction were strong enough to mask the potential positive effects ascribed to resveratrol. Consequently, a lower degree of energy restriction was preferred in the present study.

In the present study, when the effects observed in both restricted groups (R and RR) were compared, no significant differences were appreciated between them. This suggests that resveratrol is not effective in reducing liver triacylglycerols when it is administered together with a restricted diet. Similarly, no differences in the improvement of glycemic control were observed between both experimental groups, as previously reported by our group. It is interesting to point out that resveratrol behaviour is different depending on the feeding pattern, because, as it has been widely reported, this polyphenol is effective in terms of liver triacylglycerol reduction when administered in a scenario of overfeeding. “On the other hand, an important message is that resveratrol is not able to increase the effects induced by energy restriction, and consequently no additive effects were found”.

In conclusion, the present results show that resveratrol administration is useful for liver steatosis treatment in the framework of a balanced diet, although its effectiveness is lower than that of a mild energy restriction. By contrast, resveratrol is not able to increase the reduction in hepatic triacylglycerol content induced by energy restriction. Consequently, our initial hypothesis was not confirmed. The mechanisms of action mediating the effects of these two treatment strategies are very similar but not exactly the same.

## Figures and Tables

**Table 1:** Body weight gain, liver weight, hepatic triacylglycerol (TG) content, liver cholesterol (Chol) content and serum triacylglycerol, alanine aminotransferase (ALT) and aspartate aminotransferase (AST) concentrations of rats fed on the experimental diets for six weeks, and then fed a standard diet (C), or a standard diet supplemented with resveratrol (RSV), or submitted to energy restriction and fed a standard diet (R) or submitted to energy restriction and fed a standard diet supplemented with resveratrol (RR) (*n* = 9/group) for additional six weeks.

	*C*	RSV	*R*	RR	ANOVA
**Body weight gain (g)**	40 ± 4 ^a^	46 ± 5 ^a^	18 ± 4 ^b^	16 ± 2 ^b^	*p* < 0.001
**Liver weight (g)**	10.6 ± 0.2	11.4 ± 0.4	10.7 ± 0.4	11.0 ± 0.3	NS
**Hepatic TG (mg/g tissue)**	42.6 ± 4.7 ^a^	32.4 ± 3.5 ^b^	18.5 ± 2.5 ^c^	19.7 ± 1.8 ^c^	*p <* 0.05
**Hepatic Chol (mg/g tissue)**	5.3 ± 0.3 ^a^	4.2 ± 0.3 ^bc^	3.5 ± 0.5 ^c^	4.6 ± 0.3 ^ab^	*p <* 0.05
**Serum TG (mg/dL)**	68.2 ± 13.3 ^a^	56.7 ± 11.0 ^a^	39.6 ± 8.6 ^b^	43.8 ± 4.9 ^b^	*p <* 0.05
**ALT (U/L)**	31.2 ± 3.0	31.5 ± 6.6	24.0 ± 2.7	32.7 ± 5.4	NS
**AST (U/L)**	51.5 ± 3.1	57.6 ± 7.1	47.7 ± 8.1	49.0 ± 15.9	NS

Values are mean ± SEM. Differences among groups were determined by using one-way ANOVA followed by Newman Keuls post-hoc test. Values not sharing a common letter are significantly different (*p* < 0.05). NS: Not significant.
